# Abnormal Times and Their Burdens

**Published:** 1916-01-29

**Authors:** 


### Abnormal Times and their Burdens.

At the quarterly meeting of the Governors of the Leicester Royal Infirmary the report of the Board disclosed the burdens which all institutions will be called upon to bear in the abnormal and eventful year through which we have just passed. The difficulties of administration in the partial loss on military service of the honorary medical staff, the difficulty of securing residents, the loss of large numbers of trained nurses, and the enormously increased prices of all purchasable commodities were submitted. Notwithstanding these difficulties, the number of patients in the institution, civil and military, increased from 4,003 in 1914 to 4,448 in 1915. Three hundred and eighty of these patients were reported to be young men of military age, refused admission to the Services on account of physical disabilities, who had been admitted to the institution for remedial operative treatment.

The total income of the institution for the year was about £30,000. Gratifying increases in the contributions from donations, which advanced by upwards of £300, and in the contributions to the Saturday Hospital Society, were reported. The latter Fund continued its progressive career, and a record income of £16,400 was reported; of this amount £11,000 was the proportion receivable by the infirmary, compared with £10,624 in 1914. Legacies amounting to £1,790 were received during the year, and were invested in 4½ per cent. War Loan, a course which commended itself to the Governors as one which should be followed in the future with regard to all legacies. The expenditure of the institution increased with a bound to £29,150. The cost of provisions advanced by £1,900, an increase of 30 per cent. on the figures of the previous year. While the increased number of patients received accounts for approximately half of this increase, increased prices were the cause of the balance.

Salaries and wages showed an increase of £1,200, £500 of which was for the additional salaries to resident medical officers, and to certain financial arrangements which were made with medical men for carrying on the work during the year. Additions to the salaries of the nursing staff substantially accounted for the balance.

### National Services and their Penalty.

The record of this hospital not only typifies the great national service rendered by the voluntary hospitals during the year; but the enormous penalty which these institutions have to pay for the abnormal period through which the country is passing. Experience has shown, however, that income is still forthcoming, and we hope that the appeal to the manufacturers of Leicestershire in the prosperity which national demands on their output have brought to them will be generously responded to. Colonel Bond indicated that the town had a good investment for its contributions if the infirmary treated 41,000 accident cases yearly.

